# Homocysteine‐Lowering Treatment and the Risk of Fracture: Secondary Analysis of a Randomized Controlled Trial and an Updated Meta‐Analysis

**DOI:** 10.1002/jbm4.10045

**Published:** 2018-03-24

**Authors:** Maria Garcia Lopez, John A Baron, Tone K Omsland, Anne Johanne Søgaard, Haakon E Meyer

**Affiliations:** ^1^ Department of Clinical Endocrinology Morbid Obesity and Preventive Medicine Oslo University Hospital Oslo Norway; ^2^ Department of Community Medicine and Global Health Institute of Health and Society University of Oslo Oslo Norway; ^3^ Department of Epidemiology Geisel School of Medicine at Dartmouth Lebanon NH USA; ^4^ Department of Medicine University of North Carolina School of Medicine Chapel Hill NC USA; ^5^ Division of Mental and Physical Health Norwegian Institute of Public Health Oslo Norway

**Keywords:** FRACTURE, HIP FRACTURE, FOLIC ACID, VITAMIN B12, RANDOMIZED CONTROLLED TRIAL

## Abstract

High plasma homocysteine is a risk factor for osteoporotic fractures. Several studies have assessed the possible preventive effect of homocysteine‐lowering B‐vitamin treatment on the risk of fracture with inconclusive results. In the current study, we include new results from the Aspirin Folate Polyp Prevention Study (AFPPS) together with an updated meta‐analysis of randomized controlled trials (RCTs). Our objective was to determine whether there is an association between homocysteine‐lowering B‐vitamin treatment and the risk of fracture. The AFPPS trial was performed between 1994 and 2004 in nine clinical centers in the United States, and 1021 participants were randomized to a daily folic acid dose of 1 mg (*n* = 516) or placebo (*n* = 505). The main outcome was fracture of any type. In addition, we analyzed the risk of hip fracture. In the meta‐analysis, studies were identified following a search strategy in electronic database and by hand searching. Risk ratio with 95% confidence interval (CI) was chosen for pooled analyses. In the AFPPS, no statistically significant association was found between folic acid treatment and fractures of any type (risk ratio [RR] = 0.95; 95% CI 0.61–1.48) or hip fracture (RR = 0.98; 95% CI 0.25–3.89). In the meta‐analysis, six RCTs were included with a total of 36,527 participants. For interventions including folic acid and/or vitamin B12, the pooled RR for treatment was 0.97 (95% CI 0.87–1.09) for fractures of any type (*n* = 1199) and 1.00 (95% CI 0.81–1.23) for hip fractures (*n* = 335). In conclusion, no association was found between homocysteine‐lowering treatment with B vitamins (folic acid and vitamin B12) and the risk of fracture. © 2018 The Authors. *JBMR Plus* is published by Wiley Periodicals, Inc. on behalf of American Society for Bone and Mineral Research.

## Introduction

Osteoporotic fractures cause serious health problems affecting patients’ quality of life and lead to high demand for health care resources. Hip fracture especially can be devastating for the patient because it is associated with both increased morbidity and mortality and risk of recurrent fractures.^1^, ^2^ In the United States, there is evidence that the hospitalization burden of osteoporosis fractures in women aged >55 years is higher than myocardial infarction, stroke, or breast cancer, separately.^3^

Previous publications have reported a significant positive association between elevated total plasma homocysteine (tHcy) and risk of fractures.^4^, ^5^ B vitamins lower tHcy levels as they play an important role in its metabolism.^6^ Several trials have analyzed the possible effect of homocysteine‐lowering B‐vitamin treatment to prevent osteoporotic fractures.^7^, ^8^, ^9^, ^10^ Results from most of these studies have failed to show any association, except for a recently retracted Japanese trial, which reported a reduction in the risk of hip fracture in patients treated with a combination of folic acid and vitamin B12.^11^, ^12^ Two published meta‐analyses on the possible effect of B‐vitamin treatment on fracture risk have included randomized controls trials (RCTs). One included only one RCT in addition to observational studies,^13^ and the other included the retracted article described above.^14^

In this current article, we summarize two main analyses: 1) a secondary analysis of the Aspirin Folate Polyp Prevention Study (AFPPS), in which we assessed the effect of folic acid supplementation on the risk of fracture, and 2) a meta‐analysis of RCTs of the effect of homocysteine‐lowering B‐vitamin treatment on fracture risk. The AFPPS is of particular interest because it seems to include a population sensitive to *adverse* effects of folic acid supplementation, showing suggestive increases in the risk of adenomas and prostate cancer.^15^

## Materials and Methods

### Aspirin Folate Polyp Prevention Study (AFPPS)

#### Study design, randomization, and intervention

Briefly, the AFPPS was a randomized, placebo‐controlled, double‐blind clinical trial performed from July 1994 until October 2004 in nine clinical centers in the United States. Participants were patients with a recent history of colorectal adenoma. The US began folate fortification early during the recruitment period, and consequently the trial was conducted among individuals who were generally folate‐replete.

The study had a 3×2 factorial design, with an aspirin intervention (325 mg/d, 81 mg/d, or placebo daily) and a parallel intervention with folic acid (1 mg or placebo daily). The primary outcome of both interventions was the occurrence of one or more colorectal adenomas. The study design and results have been reported in more detail elsewhere.^15^, ^16^ In the present article, we focus on the folic acid intervention. In the original study, there was no effect of the folic acid intervention on the primary endpoint colorectal adenoma risk, although there were indications of increased risk in secondary endpoints.^15^

Originally, the trial was designed to study only aspirin, but shortly after enrollment began, it was expanded to examine folic acid as well; 100 individuals were randomized to aspirin or placebo before the folic acid component was initiated. Participants continued both study treatments until a surveillance colonoscopy anticipated approximately 3 years after the qualifying examination. Those who had this examination were asked to continue randomized placebo/folic acid treatment until their next surveillance colonoscopy, anticipated 3 to 5 years later.

Participants who declined to continue study treatment were followed observationally.

At enrollment, participants completed a questionnaire including information on personal features, lifestyle, and medical history. Plasma folate and plasma homocysteine levels were determined at baseline and after the first 3‐year follow‐up (more details in Cole and colleagues^15^). Plasma cobalamin (vitamin B12) and plasma pyridoxal (vitamin B6) were only determined at baseline.

#### Follow‐up and fracture events

Information on major medical events was retrieved from questionnaires sent every 4 months to participants taking study treatment, or annually during observational post‐treatment follow‐up through December 31, 2006. Compliance to the intervention was 80%, based on self‐reported information from participants. These questionnaires would have identified fractures that required hospitalization. A final telephone interview was completed between February 2010 and April 2012 to update medication use and medical events since the previous contact as well as all fractures that occurred since the start of participation in the study. Reports of important medical episodes (including fractures) were verified with blinded medical record review. Sites for fractures included finger, hand, wrist, distal forearm, elbow, arm, shoulder, rib, spine‐vertebrae, hip, knee, leg, ankle, foot, toe, and any other site (denoted as “other”). We defined a “fracture of any type” variable, which included all first fractures sustained by participants from inclusion in the trial throughout follow‐up. In addition, hip fracture was analyzed as a separate outcome and counted even if the participants had had another type of fracture previously.

#### Follow‐up and statistical analysis

Follow‐up time for the current study was defined as the period between randomization and first fracture or end of follow‐up (defined as the last contact date for participant without fracture). Incremental changes in folate levels were tested using a two‐sample *t* test. To study the association between treatment and fracture, we used generalized linear models with a log link and binomial distribution to estimate risk ratios with 95% confidence intervals (CIs).

#### Ethics

All participants gave written informed consent to treatment and to follow‐up. Human subjects committees at the clinical centers approved the study protocol. (Trials registration: http://Clinicaltrials.gov; identifier: NCT00272324.)

### Meta‐analysis

#### Search strategy

Based on a predefined protocol, search for randomized controlled trials was performed in MEDLINE (Ovid, 1946 to February 2017), CENTRAL (Cochrane Central Register of Controlled Trials, 1960 to January 2017), and EMBASE (1947 to February 2017). We also performed an additional search in the reference abstracts list for presentations at the American Society for Bone and Mineral Research (ASBMR) meetings from 2000 to 2016 and a manual search of reference lists in all relevant publications. We used MeSH (Medical Subject Headings) terms and free text words including vitamin B12 (vitamin B12, cyanocobalamin), vitamin B6 (vitamin B6, pyridoxine, pyridoxal), folic acid (folic acid, folate), homocysteine (homocysteine), fractures (fractures, osteoporotic fractures, hip fractures), and randomized controlled trial (randomized controlled trial, clinical trials, clinical study).

#### Selection criteria (eligibility)

**Figure 1:**
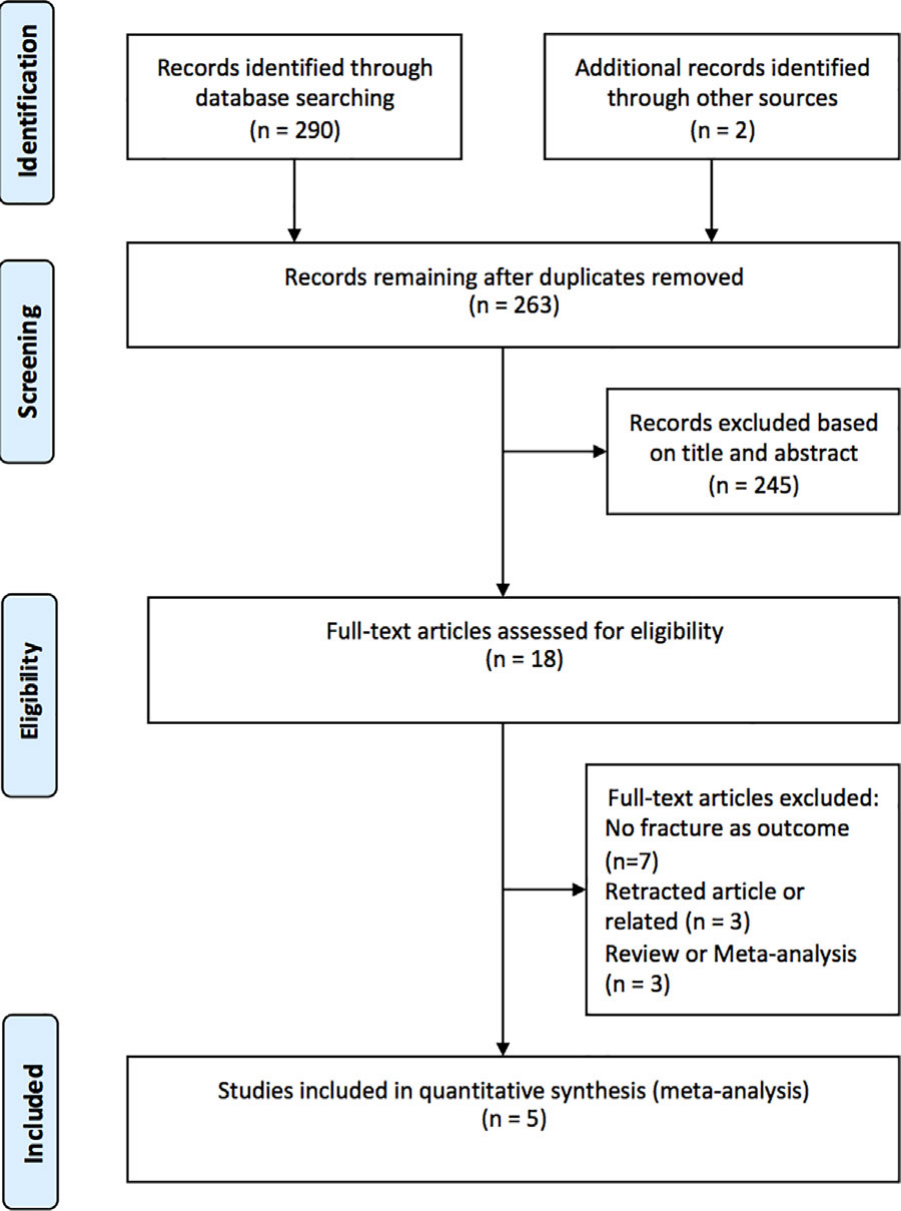
Meta‐analysis. PRISMA 2009 flow diagram.

Studies included in this meta‐analysis met the following criteria: 1) double‐blind randomized control study; 2) adult study population; 3) intervention with folic acid and/or cobalamine (vitamin B12) and/or vitamin B6 compared with placebo; 4) outcome: fractures during the trial or extended follow‐up period. If there was any doubt on eligibility based on the title and abstract of any study, the full version of the article was carefully reviewed (Fig. 1).

An abstract of the Women's Antioxidant and Folic Acid Cardiovascular Study (WAFACS) describing an intervention with folic acid and vitamin B12 was not included in the final meta‐analysis because it was only performed in a female population and it has only been reported in abstract form.^17^ However, it was included in a sensitivity analysis.

#### Outcome

We assessed two different endpoints in our meta‐analyses: fractures of any type and hip fracture. Some studies reported all fractures, whereas others reported only osteoporotic fracture (a term that was defined in each study, with slight differences in its definition between studies). Hence, we decided that a fracture variable should include all the fractures reported in each trial.

#### Assessment of risk of bias in the trials

We assessed the risk of bias in the RCTs based on the main criteria in the *Cochrane Handbook for Systematic Reviews of Interventions*: random sequence generation, allocation concealment, blinding (of participants and personnel), incomplete outcome data, selective reporting, and other bias (Supplemental Table S1).^18^ Each of the studies was then classified based on the Cochrane recommendations as having a “low” risk of bias (unlikely that bias could seriously alter the results), a “medium” risk (bias that raises some doubts about the results), or a “high” risk (bias that seriously weakens confidence in the results).

#### Statistical analysis

Results included for each RCT come from intention‐to‐treat analyses of first events (ie, fractures). In the meta‐analyses, the results were expressed as risk ratios (RR) calculated by dividing the risk of fracture in the intervention group by that in the control group. The risks were calculated by dividing the number of subjects who suffered a fracture by the total number of subjects in the group.

Heterogeneity was analyzed with the I^2^ statistic, with values <25%, 25% to 50%, and >75% considered as indicators of low, moderate, and high heterogeneity, respectively.^19^, ^20^ Publication bias was tested using Egger and Begg's regression tests and funnel plots. We also performed additional meta‐analyses adding the results from the WAFACS abstract^17^ on fracture of any type and hip fracture.

In addition, we carried out the meta‐analyses excluding studies (or study arms) co‐supplementing with vitamin B6. This decision was based on the results reported in a secondary analysis of combined data from two similar Norwegian RCTs showing an *increased* risk of hip fracture in participants treated with high doses of vitamin B6.^21^ This study had a 2×2 factorial design with the following treatments: 1) folic acid (0.8 mg), vitamin B12 (0.4 mg), and vitamin B6 (40 mg); 2) folic acid (0.8 mg) and vitamin B12 (0.4 mg); 3) vitamin B6 (40 mg); and 4) placebo. Hence, for the main hip fracture meta‐analysis, we used the marginal result from the Norwegian study, comparing the two groups receiving folic acid plus vitamin B12 (groups 1 and 2) versus those not receiving it (groups 3 and 4). In the meta‐analysis excluding study arms supplementing with vitamin B6, we included results from the comparison between the group receiving treatment with folic acid and vitamin B12 (group 2) with placebo (group 4).

Data were analyzed by STATA statistical software, version 14.2 (StataCorp., College Station, TX, USA). Review Manager 5.3 (the Cochrane Collaboration) was used for graphics and heterogeneity analyses in the meta‐analysis section. The results were considered statistically significant with a *p* < 0.05.

## Results

### Aspirin Folate Polyp Prevention Study (AFPPS)

The AFPPS trial included 1121 participants for the aspirin intervention and 1021 for folic acid (516 randomized to folic acid and 505 to placebo; see flow chart previously published).^15^ Mean duration of follow‐up time in the current analysis (from randomization until fracture or last contact) was 11.9 (SD 3.8) years. There were no substantial differences in personal characteristics between the two groups at baseline, except for plasma cobalamin (vitamin B12), which was slightly higher in the placebo group compared with the folic acid group (Table 1). Participants had a mean age of 57.4 (SD 9.6) years (range 29 to 79 years) and a mean body mass index (BMI) of 27.4 (SD 4.5) kg/m^2^ with higher percentage of male (63.8%) and non‐Hispanic white (85.6%) participants. Overall, 23.3% reported drinking at least one drink per day and 14.4% were current smokers.

**Table 1:** Baseline Characteristics of the Participants in the AFPPS Trial According to Intervention Group

Characteristics	Placebo (*n* = 505)	Folic acid (*n* = 516)
Age, mean (SD)	57.4 (9.5)	57.4 (9.6)
BMI (kg/m^2^), mean (SD)	27.4 (4.5)	27.5 (4.6)
Sex, male *n* (%)	321 (63.6)	330 (64.0)
Race/ethnicity,		
Non‐Hispanic white, *n* (%)	431 (85.3)	443 (85.9)
Current smoker, *n* (%)^a^	68 (13.6)	79 (15.3)
Alcohol intake		
≥1 drink/day, *n* (%)	110 (23.0)	117 (23.7)
Serum or plasma biochemical values, mean (SD)		
Total plasma homocysteine, μmol/L, mean (SD)	9.8 (2.9)	9.9 (2.9)
Plasma folate, ng/mL, mean (SD)	10.4 (7.5)	10.5 (7.9)
Serum cobalamine (vitamin B12), pmol/L, mean (SD)	346.8 (189.5)	321.9 (142.4)
Plasma pyridoxal 5′ phosphate (vitamin B6), nmol/L, mean (SD)	83.9 (89.6)	77.5 (88.5)
Vitamin supplements		
Multivitamin intake, yes, *n* (%)	191 (37.8)	176 (34.1)
Vitamin B12 supplements		
Self‐reported intake, yes, *n* (%)	184 (38.3)	177 (35.8)
Mg per day, mean (SD), (among those answering yes)	7.4 (6.8)	7.2(6.4)
Vitamin B6 supplements		
Self‐reported intake, yes, *n* (%)	184 (38.3)	177 (35.8)
Mg per day, mean (SD), (among those answering yes)	2.6 (2.6)	2.6(2.6)

BMI = body mass index.

SI conversion: To convert plasma folate to nmol/L, multiply by 2.266; plasma homocysteine to mg/L, divide by 7.397.

^a^ Current smoker defined as someone who smokes at least 1 cigarette per day and has been smoking that for at least the last year.

Median tHcy was 9.8 (2.9) μmol/L, and 14.4% participants presented with hyperhomocysteinemia (tHcy >15 μmol/L). Thirty‐six percent of the participants reported intake of multivitamin supplements at baseline. The mean intake per day from supplements was 7.3 μg and 2.6 mg of vitamin B12 and vitamin B6, respectively. After a 3‐year treatment with folic acid, there was a significant increase in mean plasma folate (*p* < 0.01) and a decrease in mean plasma tHcy (*p* = 0.02) levels between the groups (Supplemental Table S2).

During follow‐up, 73 participants suffered a first fracture of any type (36 women and 37 men). For the AFPPS analysis, we only considered first fracture. Although 11 participants suffered more than one fracture, the total number of fractures of any type was rather similar in the two intervention groups (no folic acid: 45 fractures versus folic acid: 42 fractures). Among those with a fracture, there was a higher proportion of women and a lower mean BMI compared with the rest of the participants (Supplemental Table S1). A total of 8 participants suffered a hip fracture. All hip fractures were first fractures.

As can be seen in Table 2, folic acid treatment did not affect the risk of fractures of any type (RR = 0.95; 95% CI 0.61–1.48) or hip fracture (RR = 0.98; 95% CI 0.25–3.89). There were no substantial changes in results after adjustment for age, sex, and BMI for fractures of any type (RR = 0.96; 95% CI 0.62–1.49) or for hip fracture (RR = 1.10; 95% CI 0.27–4.44).

**Table 2:** AFPPS Trial: Risk Ratio (RR) of Sustaining a First Fracture of Any Type or Hip Fracture Comparing Randomized Folic Acid Treatment Groups

		No. of first fractures per randomized group (rate per 1000 observations‐years)		
	Total no. of first fractures (rate per 1000 observations‐years)	Placebo	Folic acid	Risk ratio (95% confidence interval)
Fracture of any type^a^	73 (6.0)	37 (6.2)	36 (5.8)	0.95 (0.61–1.48)
Hip fracture	8 (0.7)	4 (0.7)	4 (0.6)	0.98 (0.25–3.89)

^a^ Includes the following categories: finger, hand, wrist, distal forearm, elbow, arm, shoulder, rib, spine‐vertebrae, hip, knee, leg, ankle, foot, toe, other.

In stratified analyses by sex, the RR of fractures of any type was 0.63 (95% CI 0.32–1.21) in men receiving treatment compared with men not receiving treatment, whereas for women, the corresponding RR was 1.33 (95% CI 0.68–2.57; *p* for interaction = 0.10). Diverging associations between treatment and fracture of any type were also suggested in analyses stratified on BMI: RR = 0.71 (95% CI 0.39–1.27) in those with BMI under the median and RR = 1.33 (95% CI 0.62–2.76) in those with BMI higher than the median (*p* for interaction = 0.48).

There was no interaction between the use of B‐vitamin supplements at baseline and folic acid intervention on fracture risk (*p* for interaction multivitamin = 0.8, and for vitamin B12 baseline supplements = 0.5).

Considering the aspirin intervention, there was similar risk of sustaining a fracture in participants receiving aspirin (81 mg or 325 mg) compared with placebo (RR = 1.06; 95% CI 0.64–1.74). There was no statistically significant interaction between aspirin and folic acid treatment assignments on fracture risk (*p* for interaction = 0.4).

### Meta‐analysis

The main characteristics of the included studies are shown in Table 3. These trials included a total of 36,527 participants (63.4% males, median age 64.9 years). Fractures were the primary endpoint in only one trial, which was performed among healthy community‐dwelling adults with elevated tHcy.^10^ The rest of the trials were performed among patients suffering from either cardiovascular disease (CVD)^7^, ^8^, ^9^ or colorectal adenomas^15^ and fractures were a secondary outcome. Three of the trials studied a combined B‐vitamin intervention (folic acid, vitamin B12, and vitamin B6), whereas the rest included folic acid treatment with or without vitamin B12.

**Table 3:** Meta‐Analysis: Characteristics of Trials Included in Primary Meta‐Analysis and Sensitivity Meta‐Analysis

						Baseline and follow‐up tHcy, μmol/L, mean (SD)		
Study author, year, country	Study name	Study population Inclusion criteria Age (years), mean (SD) Sex, male (%)	Sample size (*n*)	Intervention daily	Duration, mean (years)	Treatment group	Control group	Main result
Current study Garcia et al., 2017 (USA/Norway)	AFPPS	Recent history of colorectal adenomas Age 57 (9.6) Male (64%), white (85.6%)	Total: 1021 (T): *n* = 516 (C): *n* = 505	(T): 1 mg folic acid (C): placebo	3–5 (TR) 11.9 (FU)	9.9 (2.9) to 9.0 (2.2) (–0.9)	9.8 (2.9) to 9.2 (2.5) (–0.6)	73 fractures (8 hip) No association between folic acid and risk of fracture RR = 0.95 (95% CI 0.61–1.48)
Sawka et al., 2007^9^ (Canada)	HOPE‐2	Previous CVD or diabetes Community‐dwelling adults Age 68.8 Male (72%), white (92.3%)	Total: 5522 (T): *n* = 2758 (C): *n* = 2764	(T): 2.5 mg folic acid + 50 mg vit B6 + 1 mg vit B12 (C): placebo	5 (TR)	12.2 to 9.7 (–2.5)	12.2 to 12.9 (+0.7)	350 fractures No association between treatment and risk of fracture HR = 1.06 (95% CI 0.81–1.40)
Armitage et al., 2010^(7)^ (UK)	SEARCH	Previous history of CVD Age 64.2 (8.9) Male (83%)	Total: 12,064 (T): *n* = 6033 (C): *n* = 6031	(T): 2 mg folic acid + 1 mg vit B12 (C): placebo	6.7 (TR)	(–3.3)		Total fractures 495 Fracture incidence was similar between groups
Gommans et al., 2013^(8)^ (New Zealand)	VITATOPS	Recent stroke or transient ischemic attack Age 62.6 Male (64%), Western European (42%)	Total: 8164 (T): *n* = 4089 (C): *n* = 4075	(T): 2 mg folic acid + 25 mg vit B6 + 500 µg vit B12 (C): placebo	2.8 (TR) 3.4 (FU)	13.4 (5.0) to 14.3 (5.7) (+0.9)	13.5 (6.3) to 10.5 (4.2) (–3.1)	145 fractures (70 hip) No significant difference in fracture risk between treatment and placebo group RR = 0.86 (95% CI 0.62–1.18)
Van Wijngaarden et al., 2014^10^ (Netherlands)	B‐PROOF	Elevated homocysteine concentration (>12 μmol/L) Age 74.1 (≥65) Male (49.9%)	Total: 2919 T): *n* =1461 (C): *n* =1458	(T): 400 µg folic acid +500 µg B12 (C): placebo Both groups 600 UI D3	2 (TR) 3 (FU)	(median) 14.4 to 14.3 (–0.1)	(median) 14.2 to 10.3 (–3.9)	136 fractures (21 hip) No differences between groups HR = 0.84 (95% CI 0.58–1.21) Subgroup >80 years: lower risk in treatment group HR = 0.27 (95% CI 0.10–0.74)
Garcia et al., 2017^21^ (Norway)	NORVIT WENBIT NOREPOS	Ischemic heart disease Age 62.3 (11) Male (66.5%)	Total: 6837 2×2 design (T1): *n* =1708 (T2): *n* =1703 (T3): *n* =1705 (T4): *n* =1721	(T1): 0.8 mg folic acid + 0.4 mg vit B12 +40 mg vit B6 (T2): 0.8 mg folic acid + 0.4 mg vit B12 (T3): 40 mg vit B6 (T4): placebo	3.3 (TR) 11.1 (FU)	Folic acid treatment (T1)+(T2): 11.9 (4.5) to 9.0 (3.5) (–2.9)	No folic acid treatment (T3)+(T4): 12.0 (4.9) to 12.3 (5.2) (+0.3)	236 hip fractures No association between folic acid treatment and hip fracture; increased risk of hip fracture in the extended follow‐up in groups including vit B6 compared with those not including it HR = 1.42 (95% CI 1.09–1.83)
Bauer et al., 2011^(17)^ (USA), Abstract	WAFACS	Cardiovascular disease Age 62.5 (>42) Male (0%)	Total: 5442 (T): *n* = 2721 (C): *n* = 2721	(T): 2.5 mg folic acid + 50 mg vit B6 + 1 mg vit B12 (C): placebo	7 (FU)	12.1 (?)	12.5 (?)	597 fractures (22 hip) There was no significant effect of B‐vitamin treatment on non‐spine and hip fracture risk

tHcy = total plasma homocysteine; TR = trial; FU = follow‐up (trial+post‐trial); T = treatment; C = control; CVD = cardiovascular disease.

#### B‐vitamin treatment and risk of fractures of any type

**Figure 2:**
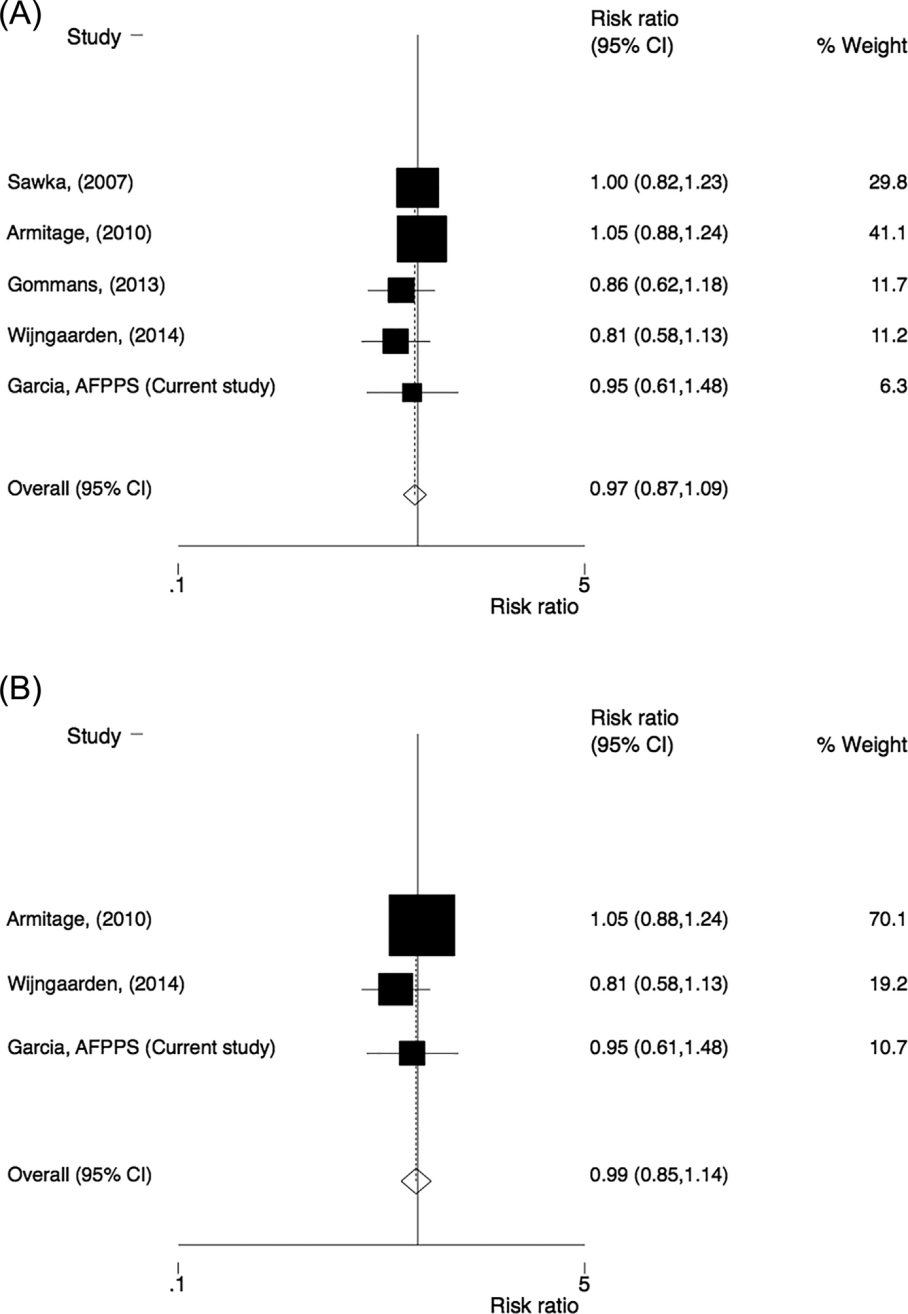
Comparison of homocysteine‐lowering treatment (folic acid with or without vitamin B12) versus placebo. Risk ratio (RR) of fracture of any type in (*A*) all studies and (*B*) after exclusion of studies co‐supplementing with vitamin B6.

In the five studies reporting data on fractures of any type, there were a total of 29,690 participants and 1236 fractures. There was no statistically significant effect of B‐vitamin treatment on the risk of fracture in the meta‐analysis (RR = 0.97; 95% CI 0.87–1.09) (Fig. 2). There was no indication of heterogeneity between the studies (I^2 ^= 0%) or publication bias (Berg's: *p* = 0.22 and Egger's test: *p* = 0.11). After exclusion of two trials co‐supplementing with vitamin B6, the estimates changed little, with RR = 0.99 (95% CI 0.85–1.14) in those treated with folic acid with or without vitamin B12 compared with placebo.

#### B‐vitamin treatment and risk of hip fracture

**Figure 3:**
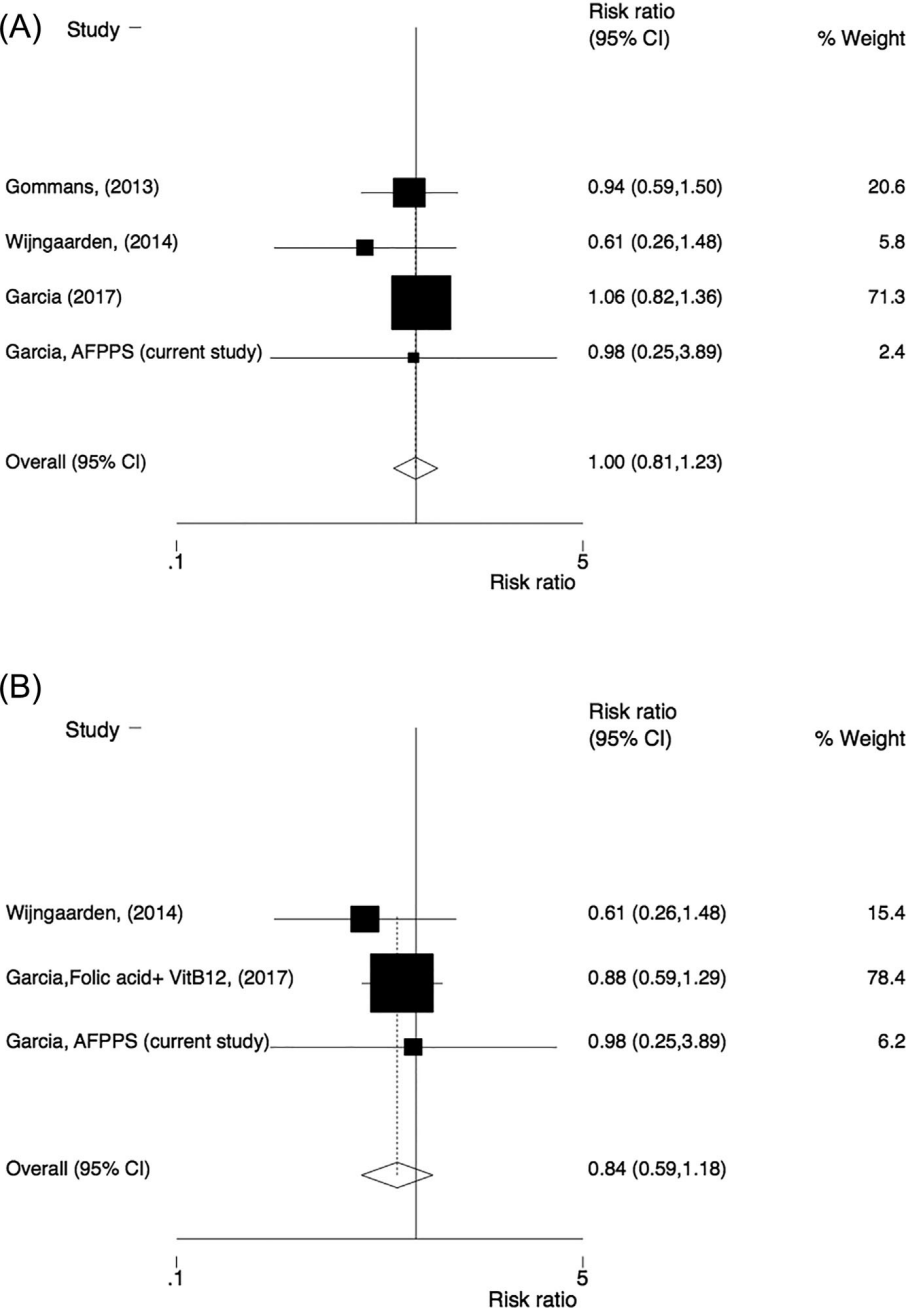
Comparison of homocysteine‐lowering treatment (folic acid with or without vitamin B12) versus placebo. Risk ratio (RR) of hip fracture in (*A*) all studies and (*B*) after exclusion of studies (or study arms) co‐supplementing with vitamin B6.

Four of the trials reported separate data on hip fracture and had a total of 18,941 participants and 326 hip fractures. There was no effect of B‐vitamin treatment on the risk of hip fracture (RR = 1.00; 95% CI 0.81–1.23). Neither heterogeneity among the studies (I^2 ^= 0%) nor significant publication bias was observed (Berg's: *p* = 0.50 and Egger's test: *p* = 0.29). Also, after excluding one trial co‐supplementing with vitamin B6, there was no statistically significant effect, with RR = 0.84 (95% CI 0.59–1.18) (Fig. 3).

#### Sensitivity analysis

Including data from the WAFAC trial, in which 5442 women received a combined intervention with folic acid, vitamin B12, and vitamin B6 over a mean of 7 years (see Table 3 for more details),^17^ did not change the results substantially, neither for all fractures (RR = 1.00; 95% CI 0.90–1.10), nor for hip fracture (RR = 1.05; 95% CI 0.85–1.30).

## Discussion

Secondary analysis of the AFPPS trial, in 1021 participants during a minimum of 3‐year and a median follow‐up of 12 years, did not show any association between the intervention with 1 mg of folic acid and the risk of fracture. These results are in accordance with our updated meta‐analyses, which included 36,527 study participants and 1236 fracture cases. There was no association between homocysteine‐lowering treatment (folic acid with or without vitamin B12) and the risk of fractures of any type or hip fracture.

Our results are in line with a previous meta‐analysis of RCTs that found no effect of homocysteine‐lowering treatment on bone turnover markers or fracture risk.^14^

Additional recent reviews that also included observational studies (with or without meta‐analysis) have reported inconclusive results on B‐vitamin treatment and bone health or fracture risk.^(6,13,22)^ Only one of them found a significant inverse association between vitamin B12 levels and fracture risk in a meta‐analysis of four observational studies.^13^ Two other reviews did not reach a final conclusion when assessing the possible effect of B‐vitamin treatment on bone health mostly because of conflicting data.^6^, ^22^ Nevertheless, all these reviews included a recently retracted trial, which introduced substantial heterogeneity to the results because it was the only RCT describing a significant strong preventive effect of B‐vitamin treatment on the risk of hip fracture.^11^

None of the RCTs included in the current meta‐analyses found an independent association between folic acid treatment and the risk of fracture, except for the B‐PROOF trial, which reported a significant reduction in fracture risk only in a preplanned subgroup analysis in individuals older than 80 years.^10^

We should also consider that the B‐vitamin doses and its combinations differed between the studies included in the meta‐analyses. The B‐PROOF study had the lower doses of folic acid (0.4 mg), though combined with vitamin B12 (0.5 mg). The HOPE‐2 and the WAFACS had the highest doses as an intervention of the three vitamins combined (2.5 mg folic acid plus 50 mg vitamin B6 plus 1 mg vitamin B12), whereas the AFPPS trial was the only one giving folic acid (1 mg) alone.

High plasma homocysteine levels may affect bone health because it leads to bone resorption (by stimulating osteoclasts’ activity) and disturbs collagen cross‐linking.^23^ Nevertheless, intervention with folic acid and/or vitamin B12 showed no reduction in fracture risk after lowering tHcy plasma levels. Except for the study reported here (AFPPS trial), the populations included in the current meta‐analyses had high mean baseline tHcy levels, either because it was a requirement for the study^10^ or because the participants had suffered a previous CVD event, a risk factor for high tHcy levels.^7^, ^8^, ^9^

All but one study were performed in countries without mandatory folate fortification. Recruitment in the AFPPS trial overlapped with the initiation of food fortification in the United States. As a result, homocysteine levels at baseline were relatively low, and it is possible that the intervention had a weaker effect in these participants who were already folate replete. On the other hand, no adverse effects on fracture risk emerged, either. However, even in trials conducted in populations with high tHcy, folic acid with or without vitamin B12 did not affect fracture risk.

The exclusion of studies or study arms including vitamin B6 did not change our conclusion, even though the RR estimate for hip fracture was somewhat lower. Thus, the lack of effect of homocysteine‐lowering treatment does not seem to be caused by co‐supplementation with vitamin B6, which has been associated with increased fracture risk.^21^ There was no possibility to perform sex meta‐analyses of the effect of homocysteine‐lowering therapies on fracture risk because most of the included studies did not provide fracture data by sex.

The WAFACS study^17^ was not included in the main meta‐analyses because it included only women and could have introduced heterogeneity to the analyses. In addition, it was only published in abstract form. However, the sensitivity analysis including it supports our findings. This secondary study added almost 600 fractures (22 of which were hip fractures). Meta‐analyses including fractures of any type and hip fractures as outcomes were both null, with little suggestion of any effect.

The AFPPS trial is a well‐designed double‐blind clinical trial. However, our study is limited by the low number of fractures because the trial was originally designed to study colorectal adenomas. In addition, some fractures were ascertained retrospectively in the final follow‐up interview. In the AFPPS trial, we did not adjust for the intake of drugs that may affect bone health. However, in a trial of this size, confounding by such covariates can reliably be excluded.

A strength of our meta‐analysis is the systematic search and that only RCTs were included, minimizing the possibility of bias. However, there are some limitations. We could not obtain detailed information about types of fractures in all trials, limiting the statistical power of the hip fracture meta‐analysis. Neither had we specific information about asymptomatic fractures from the studies, so a high proportion of vertebral fractures could have been missed. Another important limitation is that most of the studies included in our meta‐analyses had not been designed to study fracture risk as the original outcome. In addition, participants who were included had suffered a previous CVD, had a diagnosis of a colorectal adenoma, or presented a high tHcy baseline level. Therefore, we should be cautious in the generalization of our results.

In conclusion, treatment with high doses of folic acid or vitamin B12 has not shown an association with the risk of fractures. Supplementation with these vitamins is thus not recommended as a fracture‐preventive measure.

## Disclosures

All authors state that they have no conflicts of interest.

## Supporting information

Supporting Table S1.

Supporting Table S2.

Supporting Figure S1.
