# Supplementary material for: Homocysteine‐Lowering Treatment and the Risk of Fracture: Secondary Analysis of a Randomized Controlled Trial and an Updated Meta‐Analysis
Source: JBMR Plus. 2018 Mar 24;2(5):295–303. doi: 10.1002/jbm4.10045 (PMC6139704; doi:10.1002/jbm4.10045)
Supplement: Supplementary file 1 — Supporting Table S1. [file JBM4-2-295-s001.docx]

**Supp. Table 1. AFFPPS Trial. Baseline characteristics of participants according to sustaining a first fracture during follow-up.**

|  | **No fractures**  **(n=948)** | **All fractures**  **(n=73)** | ***P* value*** |
| --- | --- | --- | --- |
| Age, mean (SD) | 57.4 (9.7) | 57.7(8.3) | 0.82 |
| BMI, mean (SD) | 27.5 (4.6) | 26.3 (4.3) | **0.04** |
| Sex, male (%) | 614 (64.7) | 37 (50.6) | **0.02** |
| Race/ethnicity,  Non-Hispanic white, (%) | 809 (85.3) | 65 (89.0) | 0.39 |
| Current smoker, (%)** | 140 (14.8) | 7 (9.6) | 0.22 |
| Alcohol intake  >=1 drink/day, (%) | 211 (23.3) | 16 (23.2) | 0.98 |
| **Serum or plasma biochemical values, mean (SD)** | | | |
| Total plasma homocysteine, µmol /L | 9.91 (2.9) | 9.3 (3.0) | 0.10 |
| Plasma folate, ng/mL | 11.9 (7.6) | 11.6 (8.9) | 0.32 |
| Serum cobalamine  (Vitamin B12), pmol/L | 333.6 (170.1) | 341.2 (131.3) | 0.73 |
| Plasma pyridoxal 5’phosphate (Vitamin B6), nmol/L | 80.2 (87.9) | 87.1 (104.4) | 0.57 |
| **Vitamin Supplements** | | | |
| Multivitamin intake, yes (%) | 338 (35.7) | 29 (39.8) | 0.48 |
| Vitamin B12 supplements  - Self-reported intake, yes (%)  - mg, mean (SD),  (among those answering yes) | 332 (36.6)  7.4 (6.8) | 29 (42.0)  6.0(3.1) | 0.37  0.28 |
| Vitamin B6 supplements  - Self-reported intake, yes (%)  - mg, mean (SD),  (among those answering yes) | 332 (36.6)  2.6 (2.7) | 29 (42.0)  2.1(1.2) | 0.37  0.27 |
