# Supplementary material for: Homocysteine‐Lowering Treatment and the Risk of Fracture: Secondary Analysis of a Randomized Controlled Trial and an Updated Meta‐Analysis
Source: JBMR Plus. 2018 Mar 24;2(5):295–303. doi: 10.1002/jbm4.10045 (PMC6139704; doi:10.1002/jbm4.10045)
Supplement: Supplementary file 2 — Supporting Table S2. [file JBM4-2-295-s002.docx]

**Supp. Table 2**.

**AFPPS Trial. Incremental change in circulating plasma homocysteine and folate levels at baseline and after 3-year study treatment.**

|  | Baseline  Determination | Determination after 3 yr. | Change** | *P*-value*  Δ change |
| --- | --- | --- | --- | --- |
| **Plasma folate, ng/mL,** mean (SD) | | | | |
| Placebo group  n= 396 | 10.4 (7.5) | 13.3 (6.3) | +2.6 (8.3) | <0.01 |
| Folic acid group  n= 406 | 10.5 (7.9) | 32.9 (16.0) | +22.2 (16.9) |  |
| **Total plasma homocysteine, mg/L,** mean (SD) | | | | |
| Placebo group  n= 419 | 1.32 (0.39) | 1.24 (0.34) | -0.07 (0.3) | 0.02 |
| Folic acid group  n= 428 | 1.34 (0.40) | 1.21 (0.30) | -0.12 (0.3) |  |

* *P*-values for differences between the change in serum and plasma concentration levels (increment in change) in the different groups, were obtained using a *t*-test.

** Values do not always sum up the exact total due to rounding.
