# Supplementary figures and images for: Homocysteine‐Lowering Treatment and the Risk of Fracture: Secondary Analysis of a Randomized Controlled Trial and an Updated Meta‐Analysis
Source: JBMR Plus. 2018 Mar 24;2(5):295–303. doi: 10.1002/jbm4.10045 (PMC6139704; doi:10.1002/jbm4.10045)

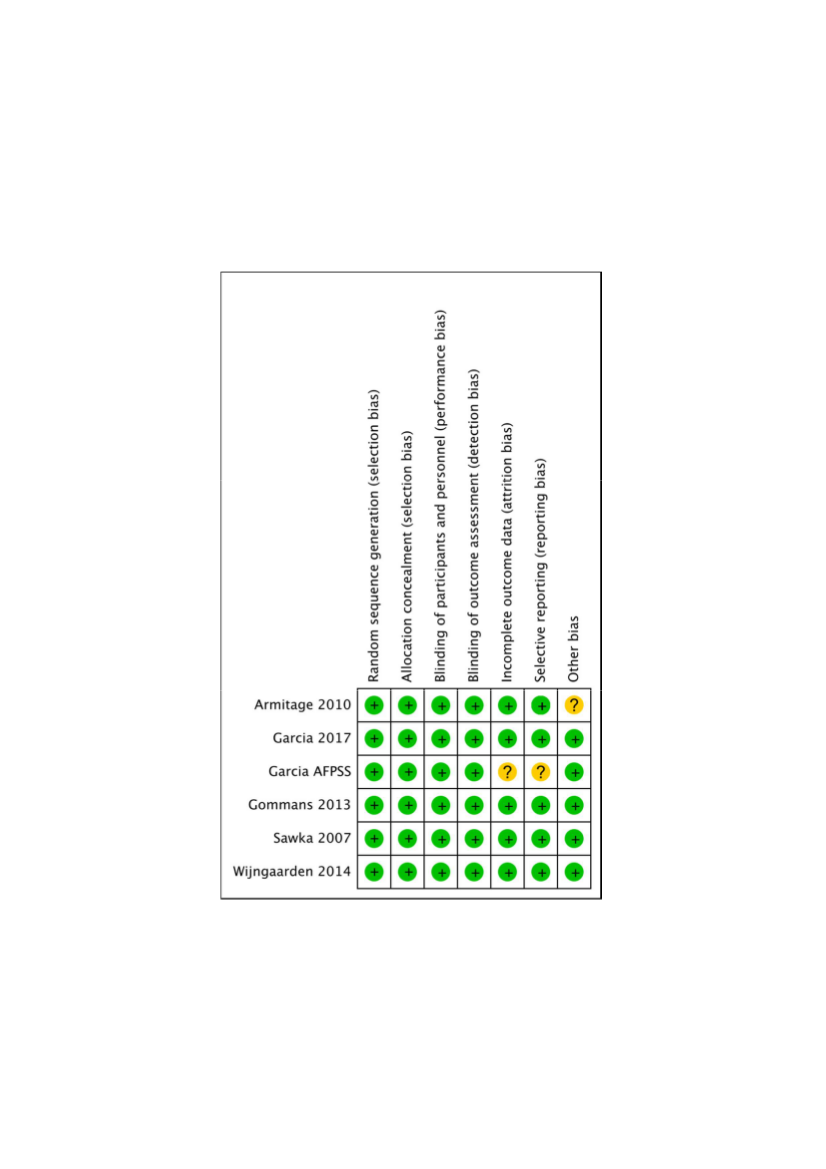

Supplement: Supplementary file 3 — Supporting Figure S1. [file JBM4-2-295-s003.tiff]
